# Genome-Wide Association Study of Gluteus Medius Muscle Size in a Crossbred Pig Population

**DOI:** 10.3390/vetsci12080730

**Published:** 2025-08-03

**Authors:** Yu He, Chunyan Bai, Junwen Fei, Juan Ke, Changyi Chen, Xiaoran Zhang, Wuyang Liu, Jing Li, Shuang Liang, Boxing Sun, Hao Sun

**Affiliations:** College of Animal Science, Jilin University, Changchun 130062, China; heyu21@mails.jlu.edu.cn (Y.H.); bcy@jlu.edu.cn (C.B.); feijw22@mails.jlu.edu.cn (J.F.); kejuan23@mails.jlu.edu.cn (J.K.); cychen24@mails.jlu.edu.cn (C.C.); xiaoran24@mails.jlu.edu.cn (X.Z.); wuyang24@mails.jlu.edu.cn (W.L.); jing_li23@mails.jlu.edu.cn (J.L.); liangshuang85@jlu.edu.cn (S.L.)

**Keywords:** pig, GWAS, gluteus medius muscle, rs81458910, *PDE4D*

## Abstract

The size of the gluteus medius muscle (GM) in swine significantly impacts both hindlimb conformation and carcass yield, while little is known about the genetic architecture of this trait. This study aims to estimate genetic parameters and identify candidate genes associated with this trait through a genome-wide association study (GWAS). A total of 439 commercial crossbred pigs, possessing both Landrace and Yorkshire ancestry, were genotyped using the Porcine 50K chip. The length and width of the GM were directly measured, and the area was then calculated from these values. The heritabilities were estimated by HIBLUP (V1.5.0) software, and the GWAS was conducted employing the BLINK model implemented in GAPIT3. The heritability estimates for the length, width, and area of the GM were 0.43, 0.40, and 0.46, respectively. The GWAS identified four genome-wide significant SNPs (rs81381267, rs697734475, rs81298447, and rs81458910) associated with the gluteus medius muscle area. The *PDE4D* gene was identified as a promising candidate gene potentially involved in the regulation of gluteus medius muscle development. Our analysis revealed moderate heritability estimates for gluteus medius muscle size traits. These findings enhance our understanding of the genetic architecture underlying porcine muscle development.

## Simple Summary

The gluteus medius muscle (GM) size in pigs affects hindlimb structure and meat yield, yet its genetic basis remains understudied. In this study, we analyzed 439 crossbred pigs to identify genetic variations associated with GM size (length, width, and area). We found these traits to be moderately heritable (with heritability ranging from 0.40 to 0.46) and identified four key genetic markers associated with the gluteus medius area (GMA). *PDE4D* was identified as a potential regulator of muscle development. These findings advance our understanding of pig muscle genetics.

## 1. Introduction

Pork constitutes a major portion of global meat consumption [1]. In response to increasing demand for animal protein [2], improving pork yield and meat quality has become a pivotal breeding goal for the pig industry [3]. The gluteus medius muscle (GM) is anatomically positioned in the proximal hindlimb region of swine and is one of the major muscles of the porcine hindlimb. The development of the gluteus medius plays a crucial role in determining the meat yield of the hindlimb and affects the quality by regulating the composition of muscle fiber types [4]. These physiological characteristics position the gluteus medius as a significant quality indicator in the processing of pork hams [5,6], thus enhancing the commercial value of the pig hindlimb. Consequently, the gluteus medius size exhibits both a direct impact on carcass yield and a significant correlation with the high-value cut percentage in swine.

Genome-wide association studies (GWAS) have emerged as a powerful strategy for elucidating the genetic basis underlying complex traits [7,8]. Currently, although an increasing number of GWAS have examined carcass quality traits, the predominant focus has remained on readily quantifiable phenotypic measures, including backfat thickness (BFT) and loin muscle area (LMA). For example, Yang et al. [9] identified the *DOCK7* gene associated with body weight and BFT utilizing GWAS in 1200 Landrace and Yorkshire pigs, and Zhuang et al. [10] localized multiple quantitative trait loci (QTLs) and genes affecting LMA and lumbar muscle depth (LMD) through GWAS in 6043 Duroc pigs. Despite these advances, the analysis of the genetic basis of deeper muscular structures such as gluteus medius is still significantly deficient. Currently, the pig QTL database (https://www.animalgenome.org/cgi-bin/QTLdb/SS/index, accessed on 26 April 2025) catalogs 3,977 gluteus medius-associated QTLs, mainly based on fat content measurements. Notably, significant gaps persist in elucidating the genetic determinants underlying gluteus medius developmental characteristics, such as gluteus medius cross-sectional area (GMA). Given the gluteus medius’s contribution to both carcass leanness and meat quality, this gap impedes our comprehensive understanding of porcine carcass quality assessment.

In this study, we performed a GWAS of GMA utilizing a population of 439 crossbred pigs which possessing both Landrace and Yorkshire ancestry. We aim to explore genetic markers governing gluteus medius development, thereby advancing marker-assisted selection strategies for lean meat production.

## 2. Materials and Methods

### 2.1. Phenotypic Data

In this study, phenotypic data were obtained from 439 crossbred pigs. These pigs were derived from a Landrace × Yorkshire rotational crossbred commercial population. All animals were raised under standardized environmental and management conditions. Following industry-standard slaughter protocols, carcass weights were systematically recorded during processing.

**Figure 1:**
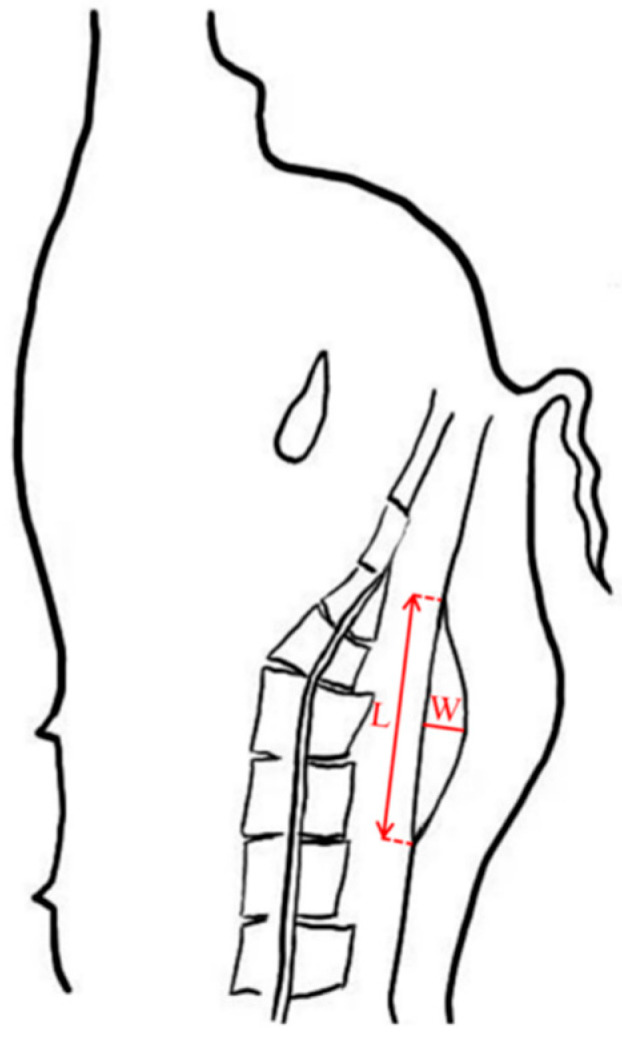
Schematic representation of the gluteus medius anatomical location and measurement protocol. L represents gluteus medius length and W represents gluteus medius width.

For morphological characterization, the gluteus medius muscle (GM) was measured on the left side of each carcass post-evisceration (Figure 1). Two primary parameters were collected, (1) gluteus medius length (GML): Maximum longitudinal dimension (mm); (2) gluteus medius width (GMW): Perpendicular width at the muscle’s broadest cross-section (mm). The cross-sectional area of the gluteus medius (GMA) was subsequently estimated, using the following formula:

(1) $$GMA=(GML\times GMW)/2({mm}^{2})$$

### 2.2. Genotypic Data

Muscle tissue samples were collected from each pig and stored in 75% ethanol at -20 °C before genomic analysis. Genomic DNA was extracted from each sample using standard phenol-chloroform extraction protocols, with nucleic acid purity assessed spectrophotometrically through absorbance ratios (A260/280 and A260/230). High-quality DNA samples were subsequently genotyped using a 50K SNP chip, yielding a total of 52,000 SNPs. Following initial genotyping, genotype imputation was performed using BEAGLE v5.4 [11] to address missing data points. Quality control (QC) was implemented through VCFtools [12] with the following exclusion criteria: (1) SNPs with minor allele frequency (MAF) < 0.05 %; (2) SNPs with call rates < 90%; (3) All non-autosomal SNPs. After QC, a total of 45,487 SNPs were retained for subsequent analysis.

### 2.3. Estimation of Genetic Parameters

The genetic parameters of GML, GMW, and GWA were estimated using a mixed linear model using HIBLUP (V1.5.0) software [13], and the analysis model was as follows:

(2) y=Xb+Za+e

where ***y*** is the vector of observed phenotypic values, ***b*** is a vector of fixed effects (carcass weight), ***a*** is a vector of additive genetic effects, assumed to follow ***a***~***N (0, G***$\sigma_{a}^{2}$***)***, where ***G*** is the genomic relationship matrix and $\sigma_{a}^{2}$ is the additive genetic variance; ***X*** and ***Z*** are the incidence matrices for fixed and random effects, and ***e*** is the vector of residual errors, with ***e***~***N(0, I***$\sigma_{e}^{2}$***)***, where ***I*** is the identity matrix and $\sigma_{e}^{2}$ is the residual variance.

### 2.4. Genome-Wide Association Study

We conducted GWAS using the BLINK model (Bayesian-information and Linkage-disequilibrium Iteratively Nested Keyway), implemented in GAPIT (version 3.0) [14], due to its high computational efficiency and strong statistical performance. The BLINK method conducts two fixed effect models and one filtering process [15]. The two fixed effect models can be written as follows:

(3) $$y_{i}=S_{i1}^{*}b_{1}+S_{i2}^{*}b_{2}+\ldots +S_{ik}^{*}b_{k}+S_{ij}d_{j}+e_{i}$$

(4) $$y_{i}=S_{i1}^{*}b_{1}+S_{i2}^{*}b_{2}+\ldots +S_{ik}^{*}b_{k}+e_{i}$$

where $y_{i}$ represents the observation on the ith individual; $S_{ik}^{*}$ is the genotype of the ***k*** pseudo QTN; $b_{k}$ is the effect of the ***k*** pseudo QTN; $S_{ij}$ is the genotype of the ith individual and jth genetic marker; $d_{j}$ is the effect of the jth marker. The optimization is performed using Bayesian information criteria (BIC). In addition, the first three principal components (PCs) were used to account for population stratification. The genome-wide significance level was defined as *p* = 0.05/N [16], where N represents the number of SNPs analyzed.

### 2.5. Functional Annotation of Significant Loci

Significant SNPs and candidate genes were mapped and annotated using the following bioinformatics resources: SNP positions were determined based on the Sus scrofa reference genome assembly 11.1 (http://ensembl.org/Sus_scrofa/Info/Index, accessed on 7 May 2025). Candidate genes within 1Mb genomic window (±500 kb) of significantly associated SNPs were identified using the BioMart data (http://asia.ensembl.org/biomart/martview/, accessed on 7 May 2025). Functional annotation analysis of candidate genes was then performed using the DAVID database (https://david.ncifcrf.gov/, accessed on 8 May 2025), including Gene Ontology (GO) term enrichment analysis and Kyoto Encyclopedia of Genes and Genomes (KEGG) pathway enrichment analysis. SNPs and candidate genes were queried for correlated traits through the PigBiobank database [17], which integrates multi-omics data including expression quantitative trait loci (eQTLs) and phenotypic associations from the PigGTEx project. Complementary gene functional analyses were performed by GeneCards (https://www.genecards.org/, accessed on 8 May 2025).

## 3. Results

### 3.1. Phenotypic Variation and Heritability Estimates

Table 1 summarizes the descriptive statistics and genetic parameter estimates for gluteus medius length (GML), width (GMW), and area (GMA). The GMA demonstrated the greatest phenotypic variability among the measured traits, exhibiting a coefficient of variation (CV) of 36.86%. The high CV of GMA suggests potential genotype-by-environment (G×E) interactions. For the genotypic data, after quality control, a total of 45,487 high-quality SNPs were retained for downstream genetic analyses. Moderate heritability estimates (0.40~0.46) were observed for all three morphometric traits, with the GMA showing the strongest genetic component (h^2^ = 0.46 ± 0.10, *p* = 6.26 × 10^−6^). All heritability estimates reached highly significant levels (*p* < 0.001), indicating substantial genetic contributions to trait variation. Population stratification was assessed using principal component analysis (PCA), with the first three PCs incorporated in subsequent association analyses to control for potential population structure effects. The 3D PCA plot is shown in Figure S1.

### 3.2. Genome-Wide Association Study

**Figure 2:**
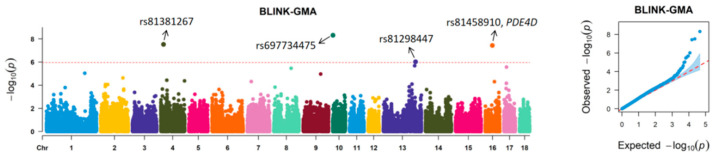
Manhattan and Q–Q plots for GWAS of the GMA trait. The solid line represents the genome-wide significant threshold of 5.96.

Given that the GMA trait exhibited the highest heritability, we subsequently conducted a genome-wide association study using the BLINK model in GAPIT3 to identify potential causal variants. Figure 2 presents the Manhattan plot and QQ plot, with the genome-wide significance threshold set at −log10(0.05/45487) = 5.96. The genomic inflation factor (λ) was calculated as 1.185. Four genome-wide significant SNPs (rs81381267, rs697734475, rs81298447, and rs81458910) were identified (Table 2). Notably, the most significant association signal was observed for rs69773447 (*p* = 4.84 × 10^−9^), located on chromosome 10, which accounted for 6.21% of the phenotypic variance. The QQ plot demonstrates the distribution of observed versus expected −log10(*p*) values, where the diagonal reference line (red) represents the null hypothesis of no association. The minimal deviation of most data points from this line indicates appropriate control of population stratification and test statistics, while the extreme outliers represent true association signals.

### 3.3. Candidate Genes Search and Functional Annotation

By integrating association results from the PigBiobank database, we annotated the top 10 phenotypic traits associated with each significant SNP (Table S1). Three SNPs (rs697734475, rs81298447, and rs81458910) showed significant associations with carcass composition and meat quality traits, including backfat thickness, lean meat percentage, and loin muscle depth. Within 500 kb flanking regions of the significant SNPs, we annotated 19 protein-coding genes (Table S2). GO and KEGG analyses suggest that these genes may be involved in energy metabolism, lipid synthesis, and signaling, thereby affecting muscle development (Table S3, Figure S2). The nearest genes to each SNP and their genomic distances are detailed in Table 2. The most significantly associated SNP, rs697734475 (chr10:1441934; *p* = 4.84 × 10^−9^), was flanked by two genes encoding regulators of G-protein signaling: *RGS21* (72.29 kb upstream) and *RGS18* (78.94 kb downstream). Furthermore, rs81381267 (chr4:14866061), accounting for 11.30% of the phenotypic variance, was positioned adjacent to metastasis suppressor 1 (*MTSS1*), which encodes an actin-binding protein crucial for cytoskeletal reorganization and cellular membrane dynamics [18]. The roundabout guidance receptor 1 (*ROBO1*) gene, located proximal to rs81298447 (chr13:176619148), encodes a receptor that binds SLIT2 ligands and mediates functions in cellular migration and angiogenesis pathways [19]. Notably, rs81458910 (chr16:39179599) accounted for 13.41% of the phenotypic variance and was located within the phosphodiesterase 4D (*PDE4D*) gene, a compelling candidate gene involved in regulating both myogenesis and adipogenesis processes.

## 4. Discussion

In modern pork production, premium meat cuts obtained through carcass segmentation (e.g., ham products derived from porcine hind limbs) significantly enhance commercial value [20]. The GM, as a principal muscular component of the hind limb, plays a crucial role in determining both limb conformation and overall carcass merit. However, due to its deep anatomical location, conventional phenotyping methods are inadequate for precise morphological assessment, necessitating the implementation of marker-assisted selection (MAS) to optimize breeding strategies.

In this investigation, we analyzed GM morphological traits (length [GML], width [GMW], and area [GMA]) in a crossbred population. Notably, the GMA exhibited the highest coefficient of variation, indicative of substantial environmental or genetic influences on this trait. Using HIBLUP for variance component estimation, we identified moderate-to-high heritability estimates (h^2^ ≥ 0.4) for all GM traits (Table 1), confirming significant genetic contributions to muscular development. Comparative analysis revealed that GML demonstrated greater heritability than GMW, suggesting that linear measurements may be more informative than transverse dimensions for the genetic evaluation of GM morphology. Importantly, GMA showed the highest heritability (h^2^ = 0.46 ± 0.10) with strong statistical significance, highlighting its exceptional potential for genetic improvement through selective breeding.

Based on these findings, we performed GWAS focusing specifically on GMA. Unlike traditional GWAS models (e.g., MLM), the BLINK algorithm employed here reduces false positives by iteratively adjusting for population structure and linkage disequilibrium [15]. The BLINK model demonstrated higher statistical power than the conventional MLM model [16]. Our crossbred population, derived from Landrace and Yorkshire lineages, enhances allele diversity and improves detection power for minor-effect loci. This genetic heterogeneity enables more robust identification of trait-associated variants within complex architectures, significantly increasing GWAS resolution. Furthermore, we employed estimated breeding values (EBVs) rather than raw phenotypic measurements for GWAS, as EBVs incorporate genomic relationships to increase detection power [21].

A total of four SNPs associated with the GMA trait were identified by GWAS. Among the identified variants, rs697734475 showed the strongest statistical significance (*p* = 4.84 × 10^−9^). The identified SNP is flanked by two members of the Regulator of G-protein Signaling (RGS) family—*RGS18* and *RGS21*. The RGS proteins regulate heterotrimeric G proteins through stimulation of guanosine triphosphatase (GTPase) activity, thereby contributing to a wide range of downstream cellular signaling [22]. GO analysis also showed that these RGS family genes are involved in G protein signaling inhibition (Table S3). *RGS18* exhibits tissue-specific expression patterns, with predominant localization in hematopoietic lineages including progenitor cells and megakaryocytes, where it plays a crucial role in modulating platelet activation and thrombotic responses [23]. In contrast, *RGS21* demonstrates ubiquitous expression across multiple tissues and participates in diverse physiological processes. In addition, several studies have revealed *RGS21* in the modulation of olfactory and gustatory perception in animals [24,25,26]. Given that chemosensory genes can influence porcine fat deposition and meat tenderness through feeding behavior modulation [27,28], we speculated that *RGS21* may indirectly regulate GM development via analogous pathways. The SNPs rs81381267 and rs81458910 accounted for 11.30% and 13.41% of the phenotypic variance, respectively, underscoring their substantial contribution to trait variability.

Notably, rs81458910 is precisely located within the *PDE4D* gene. According to GeneCards, *PDE4D* encodes a cAMP-specific phosphodiesterase that hydrolyzes the secondary messenger cyclic AMP (cAMP), serving as a critical regulator of numerous essential physiological processes. The iswine database [29] expression profile demonstrates that *PDE4D* exhibits tissue-specific expression patterns, with the highest abundance in muscle-related tissues, suggesting its crucial involvement in regulating skeletal muscle formation and function through cAMP-mediated signaling pathways. The study by Xiong et al. [30] identified a critical association between *PDE4D* and the observed variations in skeletal muscle growth across different pig breeds. Moreover, single-nucleus RNA sequencing analyses have established *PDE4D* as a molecular marker for adipocytes in both Laiwu and Large White pigs, with potential implications for intramuscular fat deposition [31,32,33]. Collectively, these findings suggest that *PDE4D* may be involved in regulating the growth and development of muscle tissues, particularly the GM, representing a novel candidate gene for porcine carcass traits and meat quality. However, studies have demonstrated that mutations in the *PDE4D* gene are associated with severe human disorders. For example, *PDE4D* was reported to be associated with asthma and acrodysostosis [34,35]. Further investigation is warranted to determine whether specific *PDE4D* mutations may increase risks of certain defects.

Integrated GO and KEGG analyses identified key genes involved in muscular lipid metabolism (Table S3). *SQLE* and *ELOVL7* were significantly enriched in endoplasmic reticulum-related GO terms and metabolic pathways (KEGG ssc01100; *p* < 0.05). SQLE (Squalene Epoxidase), encoding squalene epoxidase, catalyzes the rate-limiting conversion of squalene to 2,3-oxidosqualene in cholesterol biosynthesis. Its inhibition induces apoptosis through ER stress and altered cholesterol synthesis [36]. Differential *SQLE* expression between high- and low-lipid deposition groups in Nanyang pigs [37], along with its SNP (c.2565G>T) associations with meat quality traits [38], highlight its dual role in lipid metabolism and muscle development. ELOVL7 (Fatty Acid Elongase 7), the rate-limiting fatty acid elongase, mediates VLCFA synthesis (C18:0-C20:0) [39]. GWAS consistently associate *ELOVL7*-linked SNPs with porcine fatty acid profiles [40,41,42,43,44], supported by its downregulation in obese Yorkshire pigs [45], suggesting its importance in meat quality regulation. Additional key regulators include: RNF139 (Ring Finger Protein 139), an ER-localized E3 ligase that modulates lipid metabolism via SREBP2 processing and HMGCR degradation [46,47]; *NDUFB9* (NADH: ubiquinone oxidoreductase subunit B9), whose expression correlates with adipogenesis and is epigenetically regulated [48,49]. This integrated analysis reveals critical molecular networks governing lipid metabolism and meat quality in swine.

## 5. Conclusions

In this study, we revealed moderate heritability estimates for all gluteus medius muscle size indicators. Based on a genome-wide association study, we identified four significant SNPs (rs81381267, rs697734475, rs81298447, and rs81458910) related to the gluteus medius muscle area. Subsequent functional annotation identified two promising candidate genes (*PDE4D* and *RGS21*). These findings provide novel insights into the genetic architecture underlying porcine gluteus medius muscle size.

## Figures and Tables

**Table 1:** Descriptive statistics of phenotype and heritability of gluteus medius size.

Trait	Mean ± (SD)	Min	Max	CV (%)	h^2^ ± (SE)	*p*
GML (mm)	103.99 ± 17.74	26.86	153.83	17.06	0.43 ± 0.10	1.22 × 10^−5^
GMW (mm)	20.45 ± 5.37	4.04	35.84	26.29	0.40 ± 0.10	1.13 × 10^−4^
GMA (mm^2^)	1086.60 ± 400.56	127.10	2364.20	36.86	0.46 ± 0.10	6.26× 10^−6^

GML, gluteus medius length; GMW, gluteus medius width; GMA, gluteus medius area; SD, standard deviation; CV, coefficient of variance; h^2^, heritability; SE, standard error.

**Table 2:** Significant SNPs from the GWAS for the GMA trait.

SSC	Position	SNP ID	*p*	EPV (%)	Distance (kb)	Nearest Gene
4	14,866,061	rs81381267	2.98 × 10^−8^	11.30	67.27	*MTSS1*
10	1,441,934	rs697734475	4.84 × 10^−9^	6.21	−72.29	*RGS21*
13	176,619,148	rs81298447	9.39 × 10^−7^	9.51	−139.70	*ROBO1*
16	39,179,599	rs81458910	3.64 × 10^−8^	13.41	within	*PDE4D*

SSC, Sus scrofa chromosome; EPV, Explained phenotypic variance; Distance, the SNP located upstream/downstream of the nearest gene.

## Data Availability

Upon reasonable request, the datasets of this study can be available from the corresponding author.

## Supplementary Materials

The following supporting information can be downloaded at: https://www.mdpi.com/article/10.3390/vetsci12080730/s1, Figure S1. Three-dimensional PCA Plot. Figure S2. GO and KEGG enrichment results. Table S1. Top 10 Phenotypes Associated with Genome-Wide Significant SNPs from PigBiobank Meta-GWAS Analysis. Table S2. The distribution of genes within a 1 Mb range around significantly associated SNPs for the GMA trait. Table S3. Enrichment analysis of genes in the 1 Mb range of SNP significantly associated with GMA traits.
