# Supplementary material for: Genome-Wide Association Study of Gluteus Medius Muscle Size in a Crossbred Pig Population
Source: Vet Sci. 2025 Aug 3;12(8):730. doi: 10.3390/vetsci12080730 (PMC12389951; doi:10.3390/vetsci12080730)
Supplement: Supplementary file 1 [file vetsci-12-00730-s001.zip › Figure S2.pdf]

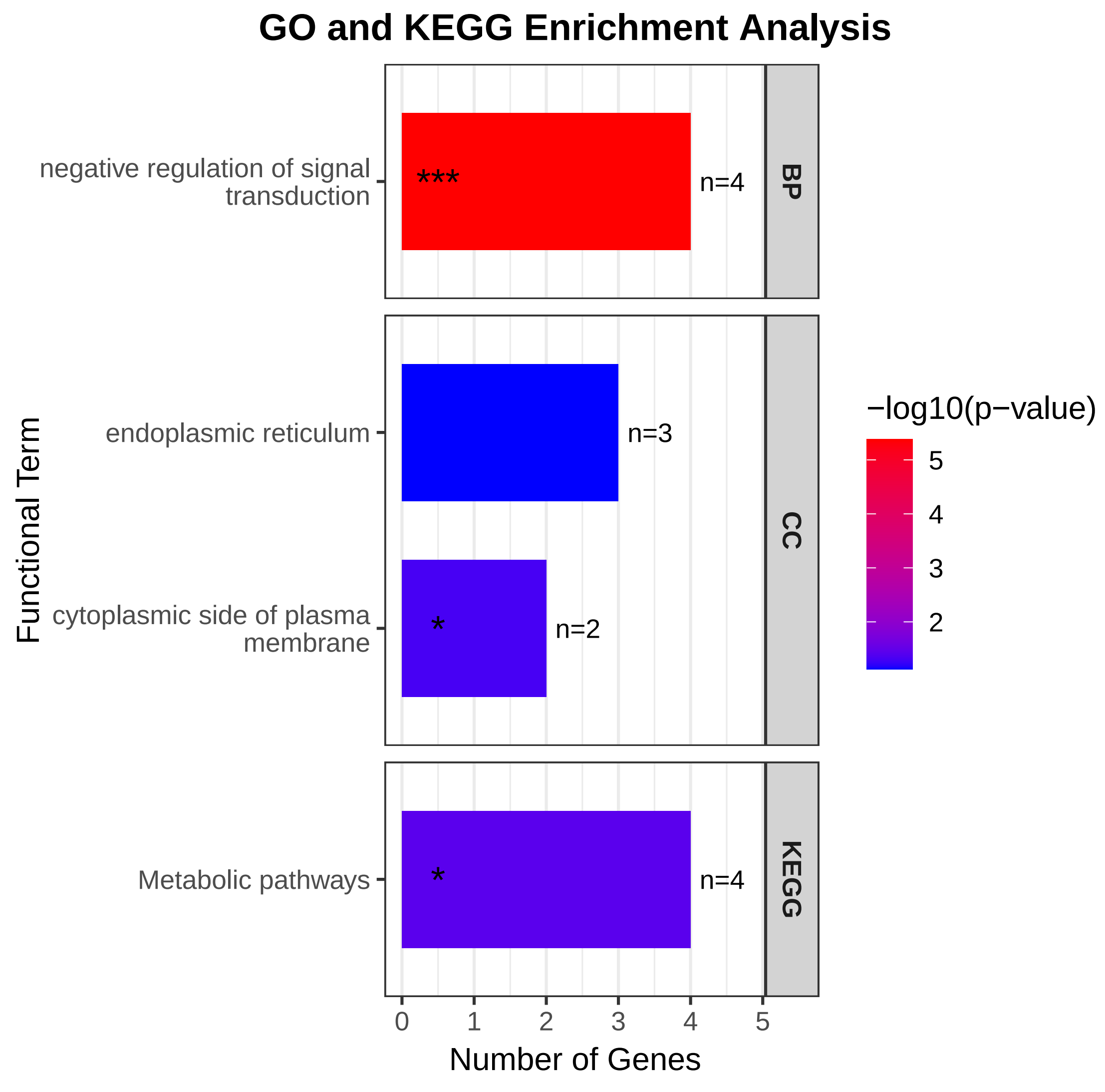
GO and KEGG Enrichment Analysis
negative regulation of signal transduction
***
n=4
BP
endoplasmic reticulum
n=3
cytoplasmic side of plasma membrane
*
n=2
CC
Metabolic pathways
*
n=4
KEGG
Functional Term
0
1
2
3
4
5
Number of Genes
−log10(p−value)
5
4
3
2
