# Supplementary figures and images for: Genome-Wide Association Study of Gluteus Medius Muscle Size in a Crossbred Pig Population
Source: Vet Sci. 2025 Aug 3;12(8):730. doi: 10.3390/vetsci12080730 (PMC12389951; doi:10.3390/vetsci12080730)

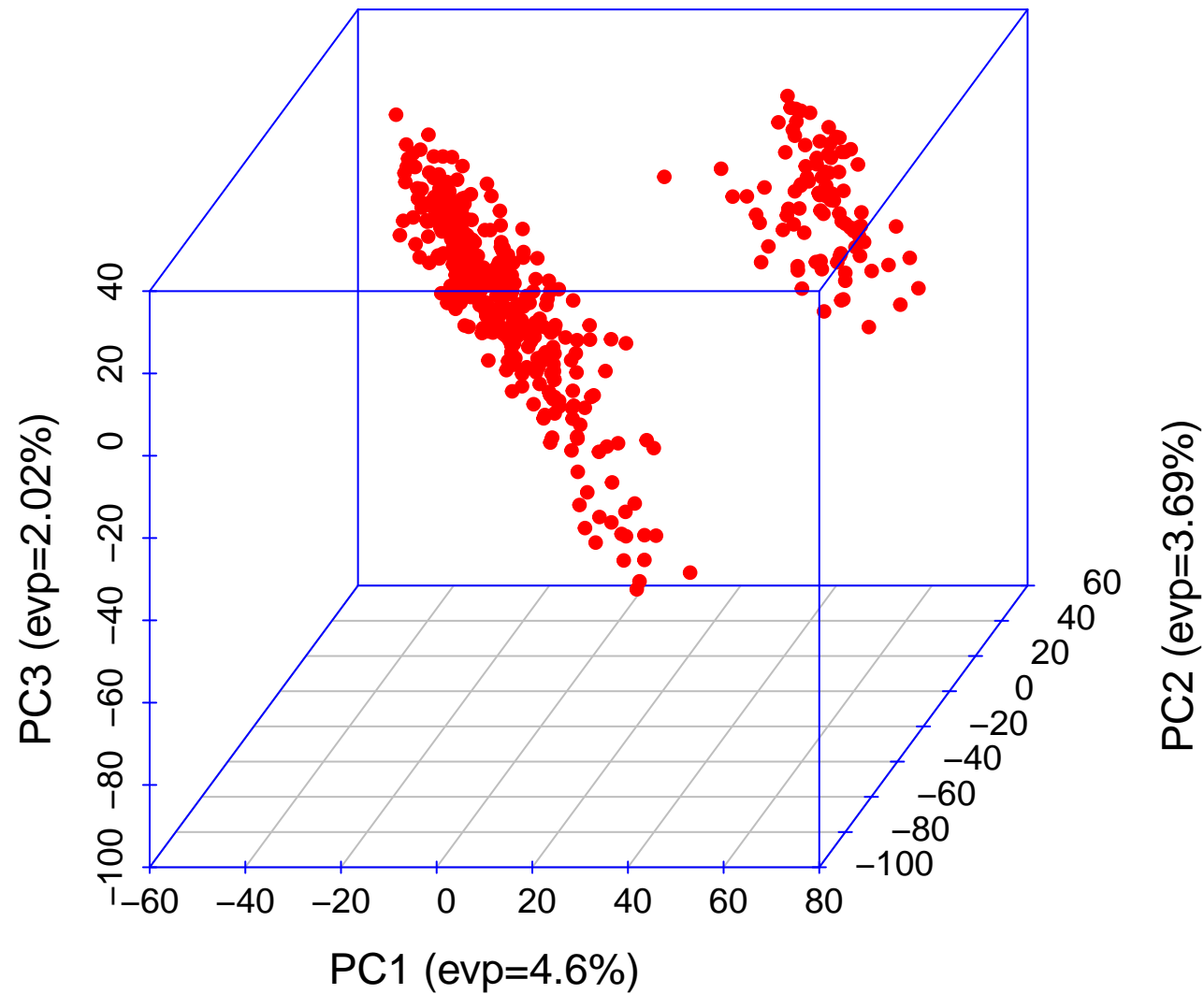

Supplement: Supplementary file 1 [file vetsci-12-00730-s001.zip › Figure S1.pdf]
